# First Dutch experience with the endoscopic laser balloon ablation system for the treatment of atrial fibrillation

**DOI:** 10.1007/s12471-014-0624-y

**Published:** 2014-11-12

**Authors:** P. Gal, J. J. J. Smit, A. Adiyaman, A. R. Ramdat Misier, P. P. H. M. Delnoy, A. Elvan

**Affiliations:** Department of Cardiology, Isala Klinieken, Dr. Van Heesweg 2, 8025 AB Zwolle, the Netherlands

**Keywords:** Atrial fibrillation, Ablation, Pulmonary vein isolation, Laser balloon ablation

## Abstract

**Introduction:**

The endoscopic laser balloon ablation system (EAS) is a relatively novel technique to perform pulmonary vein isolation (PVI) in the treatment of atrial fibrillation (AF). The present study aimed to report the results of the first 50 patients treated in the Netherlands with the EAS in terms of procedural characteristics and AF-free survival.

**Methods:**

Fifty patients successfully underwent EAS PVI. Median follow-up was 17 months. Mean age was 56 years, 82 % had paroxysmal AF.

**Results:**

99 % of the pulmonary veins were successfully isolated with the EAS. Mean procedure time was 171 min and mean fluoroscopy time was 36 min. One procedure was complicated by a temporary phrenic nerve palsy (2 %). During follow-up, 58 % of patients remained free of AF without the use of antiarrhythmic drugs.

**Conclusion:**

PVI with EAS is associated with a low risk of complications and a medium-term AF-free survival comparable with other PVI techniques.

**Electronic supplementary material:**

The online version of this article (doi:10.1007/s12471-014-0624-y) contains supplementary material, which is available to authorized users.

## Introduction

Pulmonary vein isolation (PVI) has become an important treatment modality for atrial fibrillation (AF) [1, 2] although AF recurrences can occur [3]. Several PVI techniques have been developed [4, 5] in an attempt to increase AF-free survival, among which the endoscopic laser balloon ablation system (EAS) [6, 7]. The EAS consists of a flexible, compliant balloon for sustained wall contact and an adjustable laser beam for ablation independent of tissue contact. The present study aims to report the procedural characteristics and AF-free survival after EAS PVI of the first 50 patients treated with the EAS in the Netherlands.

## Methods

Fifty consecutive patients who underwent a primo PVI using the EAS in our centre between December 2011 and December 2013 were included in a prospective registry. The prospective registry has been approved by the Institutional Review Board and all patients consented to their data being registered.

## Preprocedural care

All patients underwent transoesophageal echocardiography to rule out left atrial (LA) thrombus prior to the procedure. Patients stopped using anticoagulants and were ‘bridged’ with low-molecular-weight heparin until the day of ablation, in accordance with local guidelines.

## The endoscopic laser balloon

**Fig. 1:**
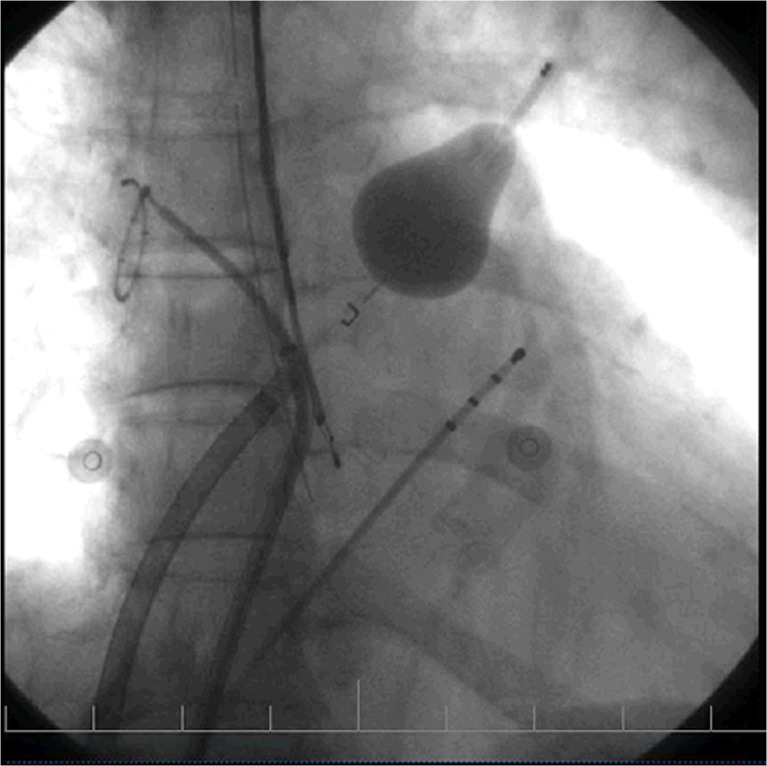
Laser balloon ablation. This figure displays the fluoroscopic setup of EAS PVI. The EAS has been inflated in the left upper PV. EAS: endoscopic laser balloon ablation system; PVI: pulmonary vein isolation; PV: pulmonary vein

The EAS (CardioFocus, Marlborough, MA, USA) is a balloon-based catheter system. Its characteristics have been described previously [7, 8]. The EAS was manoeuvred to each pulmonary vein (PV) ostium under fluoroscopic guidance (Fig. 1). A ring of atrial myocardium antral to the PV was exposed by varying the EAS balloon inflation size. Laser energy was delivered to the exposed ring of atrial tissue. After a full circle was completed, the EAS was retracted from the PV. The ablation procedure is also illustrated in an online movie (online movie). A circular mapping catheter was introduced to assess persistent electrical connection between the PV and the left atrium. If any existed, the EAS was re-introduced to the PV, and additional lesions were applied by the operator. No adenosine testing was performed. An oesophageal temperature probe (SensiTherm, St Jude Medical, USA) was inserted, and energy delivery was instantaneously terminated when the temperature exceeded 39.0 °C. During ablation of the right-sided PVs, stimulation of the phrenic nerve (using 20 mA at 2.9 ms) was performed, with immediate cessation of energy delivery once capture was diminished or lost.

## Follow-up

Patients visited the outpatient clinic at 3, 6, 12, 18 and 24 months after PVI, including 24-h Holter ECG. AF recurrence was defined in accordance with European guidelines [1]. In all patients, antiarrhythmic drugs were ceased 3 months after the PVI.

## Study endpoints

The primary endpoint of our study was AF-free survival after EAS PVI. Secondary endpoints were: acute PVI, procedure time, ablation time and fluoroscopy time. The safety endpoint was major or minor complications within 30 days of the procedure as described in European guidelines [1].

## Statistical analysis

Continuous variables were expressed as mean with standard deviation in case of normal distribution or median with interquartile range when not normally distributed. Statistical analysis was performed using IBM SPSS statistics version 20 (IBM inc., Armonk, NY, USA).

## Results

Baseline characteristics of the 50 consecutive patients are displayed in Table 1. Mean age was 56 years, 82 % had paroxysmal AF. No LA thrombi were found during the preoperative transoesophageal echocardiogram. There were 2 left-sided and 1 right-sided common PVs and there were 2 right-sided accessory PVs.

**Table 1:** Baseline characteristics

Patient characteristic	Total (*n* = 50)
Gender female (%)	28 %
Age (years)	55.9 (±10.7)
BMI (kg/m^2^)	26.9 (±3.6)
Persistent AF	18 %
AF duration (years)	7.0 (±6.5)
Failed AADs (range)	1.4 (0–4)
LA ventral-dorsal dimension (mm)	41.1 (±3.9)
LVEF (%)	58.8 (±3.2)
History of hypertension	30 %
History of diabetes mellitus	6 %

Data are presented as percentages or means ± their SD or ranges where appropriate; *BMI* body mass index; *AF* atrial fibrillation; *AADs* antiarrhythmic drugs; *LA* left atrial; *LVEF* left ventricular ejection fraction

## Ablation results

In 198 out of 199 PVs (99.5 %), acute PVI was achieved. One common left-sided PV could not be isolated due to a temperature rise of the oesophagus. One procedure was converted to radiofrequency catheter ablation after ablation of the right upper PV was complicated by a phrenic nerve palsy, which did not prolong hospital stay and had fully recovered after 6 months. This was the only complication we observed (2 %). Table 2 displays the procedural characteristics of all patients.

**Table 2:** Procedural characteristics

Procedure time (min)	170 (±40)
Ablation time (min)	61 (±28)
Fluoroscopy time (min)	36 (±10)

Data are presented as mean ± their SD

## Follow-up

After a median follow-up of 17.3 (interquartile range: 12.9–19.5) months, 58 % of patients were free of AF after a single EAS PVI without the use of antiarrhythmic drugs.

## Discussion

The present study reports the results of the first 50 patients treated with the EAS in the Netherlands. Acute PVI can be achieved virtually always, with a low risk of complications. Moreover, medium-term AF-free survival seems to be comparable with other PVI techniques.

The EAS combines a compliant balloon design, an endoscope for visualisation of PV antral tissue and a power-adjustable laser beam. Previous reports have shown that acute PVI can be achieved virtually always with the EAS, which was also observed in the present study [8, 9]. In the present study, the complication rate is low (2 %), which is consistent with previous reports [7, 9].

Although PVI is an important treatment modality for AF, the medium-term AF-free survival is still 60–80 %, after PVI using different techniques, such as radiofrequency catheter ablation [3] and cryoballoon ablation [4]. In the present study, AF-free survival after a single EAS PVI attempt was 58 %, which is in line with a previous report [9], although there are other EAS studies reporting a higher AF-free survival [10, 11]. Potentially, a learning curve may, in part, have affected the AF-free survival in the current study. A randomised trial will provide further evidence of the AF-free survival after EAS [12]. Based on the current literature, the AF-free survival rate after EAS PVI seems to be comparable with other techniques.

AF recurrences are generally regarded as recurrence of electrical conduction over the PV-LA junction [13, 14]. Durable lesion sets therefore remain pivotal in improving medium-term AF-free survival after PVI. The medium-term success percentage suggests the lesion sets created with the EAS are not persistent. This was also suggested in another study [10], which reported that 62 % of the studies patients had 4 isolated PVs 3 months after the initial EAS PVI procedure.

Future studies should be aimed at identifying factors that are associated with AF-free survival after EAS PVI. Although the EAS consists of a compliant balloon, the catheter-tissue contact may be suboptimal in some patients, limiting the operator’s ability to deliver circular, transmural lesion sets which may influence AF-free survival. Moreover, in case of insufficient occlusion or ablation near to a blood pool, ablation energy had to be reduced. One study [15] showed high-energy EAS ablation was favourable to low-energy EAS ablation in terms of persistent electrical conduction over the PV-LA junction and AF-free survival after PVI. Although highly speculative, these factors may explain the medium-term AF-free survival in the present study.

## Conclusion

The EAS is a promising technique with a high acute PVI success rate and a low risk of complications. Medium-term AF-free survival after EAS PVI is comparable with other PVI techniques.

## Electronic supplementary material

Below is the link to the electronic supplementary material.

ESM 1 (AVI 20782 kb)
